# Characterization and genome analysis of the novel virulent Burkholderia phage Bm1, which is active against pan-drug-resistant *Burkholderia multivorans*

**DOI:** 10.1007/s00705-025-06282-w

**Published:** 2025-04-16

**Authors:** Evgenii Rubalskii, Ludwig Sedlacek, Jan Hegermann, Leonard Knegendorf, Christina Salmoukas, Carsten Mueller, Nicolaus Schwerk, Dirk Schlüter, Arjang Ruhparwar, Christian Kuehn, Stefan Ruemke

**Affiliations:** 1 Department of Cardiac, Thoracic, Transplantation and Vascular Surgery, Hannover Medical School, 30625 Hannover, Germany; 2 Lower Saxony Centre for Biomedical Engineering, Implant Research and Development, 30625 Hannover, Germany; 3 Institute for Medical Microbiology and Hospital Epidemiology, Hannover Medical School, 30625 Hannover, Germany; 4 Institute of Functional and Applied Anatomy, Research Core Unit Electron Microscopy, Hannover Medical School, 30625 Hannover, Germany; 5 Department of Pediatric Pneumology Allergology and Neonatology, Hannover Medical School, 30625 Hannover, Germany

## Abstract

The escalating challenges of antibiotic resistance in bacterial pathogens have necessitated the exploration of alternative therapeutic strategies. Among these, bacteriophage therapy has regained attention as a promising approach to combat multidrug-resistant bacteria. Bacteriophages are viruses that infect and lyse specific bacterial strains, making them attractive candidates for targeted antimicrobial treatment. *Burkholderia multivorans*, a Gram-negative bacterium, is known to cause opportunistic infections, particularly in individuals with a compromised immune system or with cystic fibrosis. The prevalence of antibiotic-resistant *Burkholderia* strains has raised concerns about treatment options. In this study, we characterized the *Burkholderia* phage Bm1, a virulent bacteriophage isolated from an environmental source. Electron microscopy revealed that Bm1 phage particles have myovirus morphology, with an icosahedral head of 72 nm in diameter and a contractile tail of 100 nm in length and 18 nm in width. The genome of phage Bm1 consists of a double-stranded DNA of 67,539 bp with a terminal repeat region at each end. Comparative analysis indicated that the closest relative of phage Bm1 is *Burkholderia* phage BCSR129, with a calculated VIRIDIC identity of 57.7%. The apparent absence of an integrase gene suggests that the *Burkholderia* phage Bm1 has a strictly lytic life cycle.

**Supplementary Information:**

The online version contains supplementary material available at 10.1007/s00705-025-06282-w.

## Introduction

*Burkholderia multivorans*, a member of the *Burkholderia cepacia* complex (BCC), encompasses both environmentally and clinically significant bacteria, and its pathogenic potential is of growing concern. It is a Gram-negative bacterium that can be found in diverse ecological niches in the environment and generally exists as a saprophyte. However, *B. multivorans* has emerged as an opportunistic pathogen capable of causing severe infections, particularly in immunocompromised individuals. Of particular concern is the prevalence of *B. multivorans* in patients with cystic fibrosis, in whom chronic respiratory infections can lead to significant morbidity and mortality. Furthermore, the emergence of multidrug-resistant *Burkholderia* strains has compounded the challenges of treating *Burkholderia*-associated infections [1, 2].

Reliance on antibiotics for treating bacterial infections has contributed to the development of antibiotic resistance, necessitating the exploration of alternative therapeutic options. Bacteriophages, or phages, offer a potential avenue for targeted antimicrobial treatment. These viruses are highly specific for their bacterial hosts, recognizing surface receptors on the bacterial cell for infection. By injecting their DNA into the host cell, phages hijack the bacterial machinery for their replication and eventually lyse the cell, releasing new phage progeny. This lytic activity can be harnessed for therapeutic purposes, as bacteriophages can effectively target and destroy bacterial pathogens [3–5].

The potential of bacteriophage therapy to combat *Burkholderia* infections has been studied in recent years. Various investigations have demonstrated the efficacy of phages against BCC members in both *in vitro* and *in vivo* models [6–8]. In particular, phage therapy has shown promise in treating chronic respiratory infections in cystic fibrosis patients caused by antibiotic-resistant BCC [9–11]. The ability of phages to co-evolve with their bacterial hosts further raises the possibility of combating antibiotic-resistant BCC effectively [12].

Given the escalating need for alternative treatments to combat antibiotic-resistant bacteria, isolation and characterization of novel bacteriophages targeting *B. multivorans* has increased in importance. In this study, we present the characterization of the newly isolated virulent *Burkholderia* phage Bm1, which was obtained from an environmental source. Understanding the genetic and morphological features of this phage will contribute to its potential therapeutic application for treating *Burkholderia* infections.

## Materials and methods

### Bacterial isolates and strains

The following clinical isolates were used in the study: *B. multivorans* 5444, *B. multivorans* 5500, *B. multivorans* 5900, *B. multivorans* 3917, *B. cenocepacia* 840, *B. cenocepacia* 856, *B. cenocepacia* 902, and *B. cenocepacia* 903. *B. multivorans* isolate 5444 (strain NZPT 0170, BioProject accession no. PRJNA1172336) was isolated from a patient with cystic fibrosis at the Hannover Medical School. *B. multivorans* isolates 5500 and 5900 were isolated from the same patient 12.5 and 13.5 months later, respectively. *B. multivorans* 5444 was determined to be a pan-drug-resistant strain, using the minimum inhibitory concentration method to evaluate resistance to clinically available antibiotics (Supplementary Fig. S1). In addition, the standard reference strains *Pseudomonas aeruginosa* DSM 1117, *Escherichia coli* DSM 1103, *Staphylococcus aureus* DSM 2569, and *Enterococcus faecalis* DSM 2570 were used as controls to exclude potential contamination with commonly occurring phages of other groups and for testing the growth media. The bacteria were grown at 37°C in tryptic soy broth (TSB) (Sigma-Aldrich, Germany). The incubation time for the *Burkholderia* isolates was 42–48 hours. *P. aeruginosa* DSM 1117, *E. coli* DSM 1103, *S. aureus* DSM 2569, and *E. faecalis* DSM 2570 were cultivated for 18–24 hours.

### Bacteriophage isolation

*Burkholderia* phage Bm1 was isolated from wastewater collected in the Summer of 2018 in Hannover, Germany, filtered using a Filtropur vacuum filtration unit with a 0.22-µm PES filter (Sarstedt AG, Germany) and stored at 4°C. Phage isolation was performed using the double-layer plate method as described previously, with slight modifications [3]. Briefly, 1 mL of wastewater and 0.2 mL of a 48-hour culture of *B. multivorans* 5444 were added to 2.5 mL of soft agar consisting of Miller lysogeny broth (LB) (Sigma-Aldrich, Germany) and 0.7% agar-agar that had been melted and cooled to 49°C. The mixture was immediately poured onto plates containing LB and 1.5% agar-agar (LB-A), spread evenly, and allowed to solidify for 5 minutes. The resulting double-layer plates were incubated upside down for 24 hours at 37°C. A total of 10 samples were tested, and a single plaque was collected using a 1000-µL filtered pipette tip and suspended in 10 mL of sterile saline. A 5 µL aliquot of the first-generation Bm1 phage suspension was mixed with 0.2 mL of *B. multivorans* 5444 culture and 3.5 mL of LB soft agar containing 0.4% agar-agar and poured onto LB-A plates, which were allowed to solidify and incubated at 37°. The procedure was repeated two more times, resulting in a pure culture of the phage.

### Bacteriophage propagation

After the third passage, the phage was propagated as described above, except that 1 mL of phage suspension and LB Broth Vegitone (Sigma-Aldrich, USA) were used instead of LB. After overnight incubation, the resulting network-patterned soft agar layer was collected and suspended in 10 mL of sterile saline, followed by centrifugation in a swinging bucket rotor at 4200 × *g* for 45 minutes at room temperature. The supernatant was passed through a 0.22-µm PES syringe filter (Sarstedt AG, Germany), and the phage titer was determined using a conventional plaque assay with a series of tenfold dilutions in sterile saline.

### Host range

The lytic activity of the phage was evaluated by serial dilution spot testing, and the efficiency of plating was determined by the double layer plaque assay using the bacterial strains listed above [13, 14]. The phage titer in the undiluted phage lysate was 2 × 10^10^ PFU/mL. Both undiluted and diluted samples were applied in a volume of 10 µL.

### Phylogenetic analysis of selected bacteria

*Burkholderia* isolates were sequenced using standard BCR1 and BCR2 primers as described previously [15] to investigate the relationship between the host range of the *Burkholderia* phage Bm1 and the bacterial phylogeny. Multiple alignment of the resulting nucleotide sequences of the *recA* gene, together with publicly available *recA* gene sequences from type strains, was performed using the MAFFT algorithm [16]. Pairwise distances derived from the multiple alignment were used to construct a phylogenetic tree, rooted to *B. multivorans* isolate 5444, using the Tamura-Nei genetic distance model and the neighbor-joining method in Geneious Prime 2022 software (Biomatters Ltd., New Zealand) with 100 bootstrap replicates.

### One-step growth curve

A one-step growth curve was performed in triplicate in order to determine the latent period, the eclipse period and the average phage burst size as described [3].

### Transmission electron microscopy

A phage suspension (1.23 × 10^11^ PFU/mL) was used without dilution. A carbon film was prepared by indirect evaporation of spectral coal (Balzers) onto freshly split mica (Plano, Wetzlar, Germany) at 1.5 kV and 100 mA for 30 seconds in a Balzers EVM 052 unit. The carbon film was floated from the mica onto the phage suspension for 30 s, retracted, and then floated onto water for 5 s, retracted, and finally floated onto 6% phosphotungstic acid, from which it was picked with a 300 mesh copper grid and air dried after removal of excess fluid with wet filter paper from the side of the grid. Grids were stored dust-free and imaged on the day of preparation, using a Zeiss TEM 900 microscope (Zeiss, Oberkochen, Germany) at 80 kV with a side-mounted CCD camera (TRS, Moorenweis, Germany). Image processing included clipping, positioning, and adjustment of brightness and contrast, using Fiji [17] and Microsoft PowerPoint software.

### Whole-genome sequencing

Initial phage genome sequencing was performed using an Oxford Nanopore Technologies (ONT) MinION device to determine the overall genome structure and composition and to exclude potentially induced prophages from the host bacteria. DNA extraction was performed using a Phage DNA Isolation Kit (Norgen Biotek, Canada) with DNase I treatment according to the manufacturer's protocol, followed by library preparation and sequencing using a Rapid PCR Barcoding Kit (Oxford Nanopore, UK) and a MinION Flow Cell R9.4.1 (Oxford Nanopore, UK) according to the manufacturer's protocol.

More-precise sequencing of the phage genome was performed using Illumina technology. DNA was extracted from the phage lysate using a Phage DNA Isolation Kit (Norgen Biotek, Canada) without DNase I treatment according to the manufacturer's protocol. DNA quality was evaluated using a NanoDrop One Spectrophotometer (Thermo Fisher Scientific) and then quantified using a Qubit dsDNA High Sensitivity Assay Kit on a Qubit 4.0 Fluorometer (Thermo).

Shotgun library preparation was done using an Illumina DNA Prep (M) Tagmentation Kit (Illumina) according to the manufacturer’s instructions, with the following changes: all volumes were reduced to 1/3, using an input amount of 12 ng and six cycles for index-ligation (BLT-PCR step), and Unique Duel Index Adapters (UDP) Plate Set A (Illumina) was used.

Libraries were quality-checked using D5000 High Sensitivity ScreenTape (Agilent) on a 4200 Tape Station (Agilent), and the concentration was measured again using a Qubit dsDNA High Sensitivity Assay (Invitrogen) on a Qubit 4.0 Fluorometer. Libraries were pooled in equimolar amounts (prior normalization to 4 nM), and the library pool was sequenced on a MiSeq System V2 (2 × 250 bp) according to the manufacturer’s instructions.

### Bioinformatic analysis

The bioinformatic tools were used under the Ubuntu v.22.04.3 LTS operating system with Python v.3.10.8. Phage genome assembly based on the nanopore sequencing results was performed using the following software: base recognition was performed using Guppy software with subsequent removal of adapter sequences from the obtained reads using Porechop software [18]. Read quality control was done using NanoFilt software [19], *de novo* genome sequence assembly was done using Canu v.1.9 software [20, 21], and alignment to the assembled *de novo* sequence was done using UGENE v.45.1 software [22], using the BWA-MEM algorithm.

Paired reads from Illumina sequencing were filtered using Trimmomatic v.0.33 [23], assembled *de novo* using SPAdes assembler v.3.15.4 [24], and aligned to the assembled sequence using UGENE v.45.1 software [22] with the Bowtie2 algorithm.

The phage genome was annotated using Pharokka v.1.3.2 [25] in the Conda environment v.23.3.1. Specifically, coding sequences (CDSs) were predicted using PHANOTATE v.1.5.1 [26], tRNAs were predicted using tRNAscan-SE 2.0 [27], and tmRNAs were predicted using Aragorn v.1.2.41 [28]. Functional annotation was done by matching each CDS to the PHROGs [29], VFDB [30], and CARD [31] databases (Pharokka Database v1.2.0, https://zenodo.org/records/7563578) using MMseqs2 v.13.45111 [32] and PyHMMER v.0.9 [33]. Plots were created using pyCirclize v.0.3.1 [34]. Terminal repeat regions were resolved using Geneious Prime 2022 (Biomatters Ltd., New Zealand) software. The termini were confirmed and the packaging mechanism was determined using PhageTerm v. 1.0.12 [35].

An initial search of the standard public NCBI nucleotide databases (nr/nt, accessed on 05.02.2024) for sequences from similar phages was performed using BLASTn (NIH NCBI, USA). The full genome sequence of *Burkholderia* phage Bm1 was compared to those of its closest relatives using Virus Intergenomic Distance Calculator (VIRIDIC) [36]. Prediction of phage lifestyle was performed manually based on the genome annotation as well as using a PhageAI machine learning approach [37].

## Results and discussion

### Phage isolation and host range

**Fig. 1:**
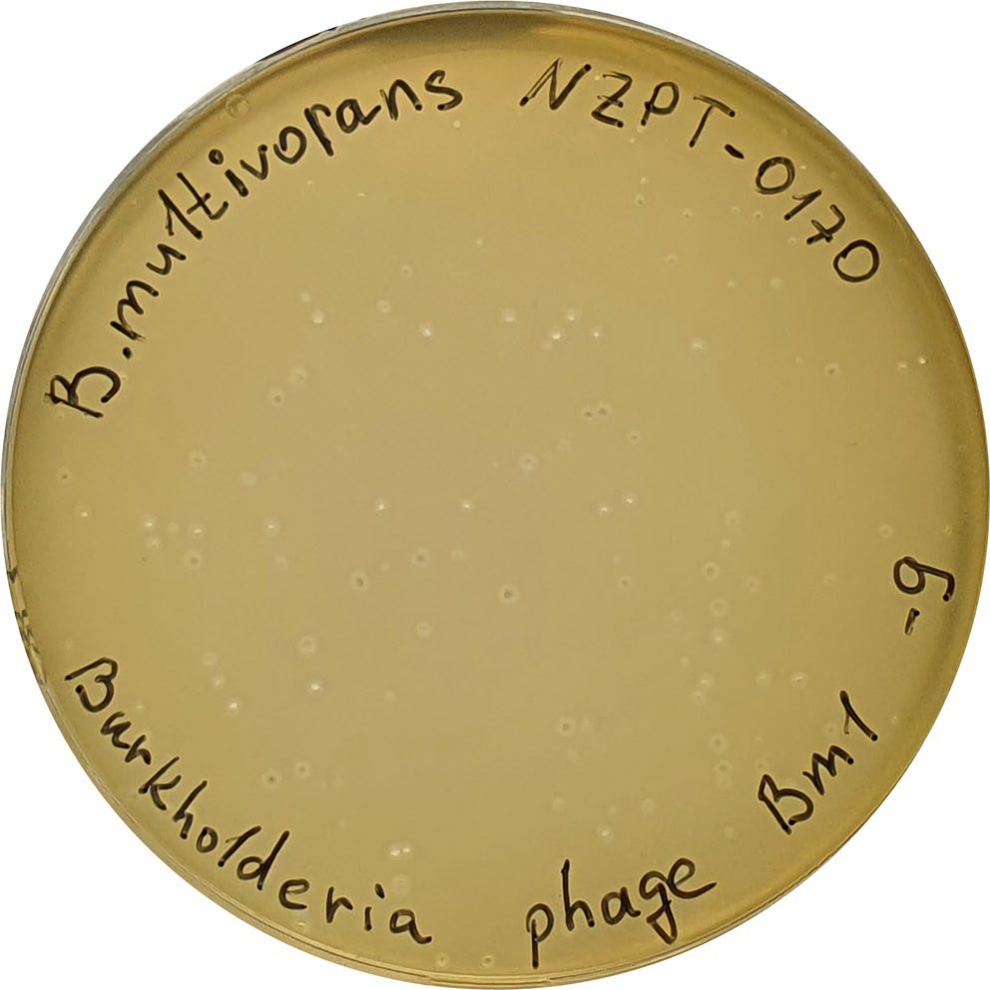
Plaques produced by phage Bm1 after incubation for 24 h at 37°C on a lawn of *B. multivorans* 5444 (NZPT 0170)

**Fig. 2:**
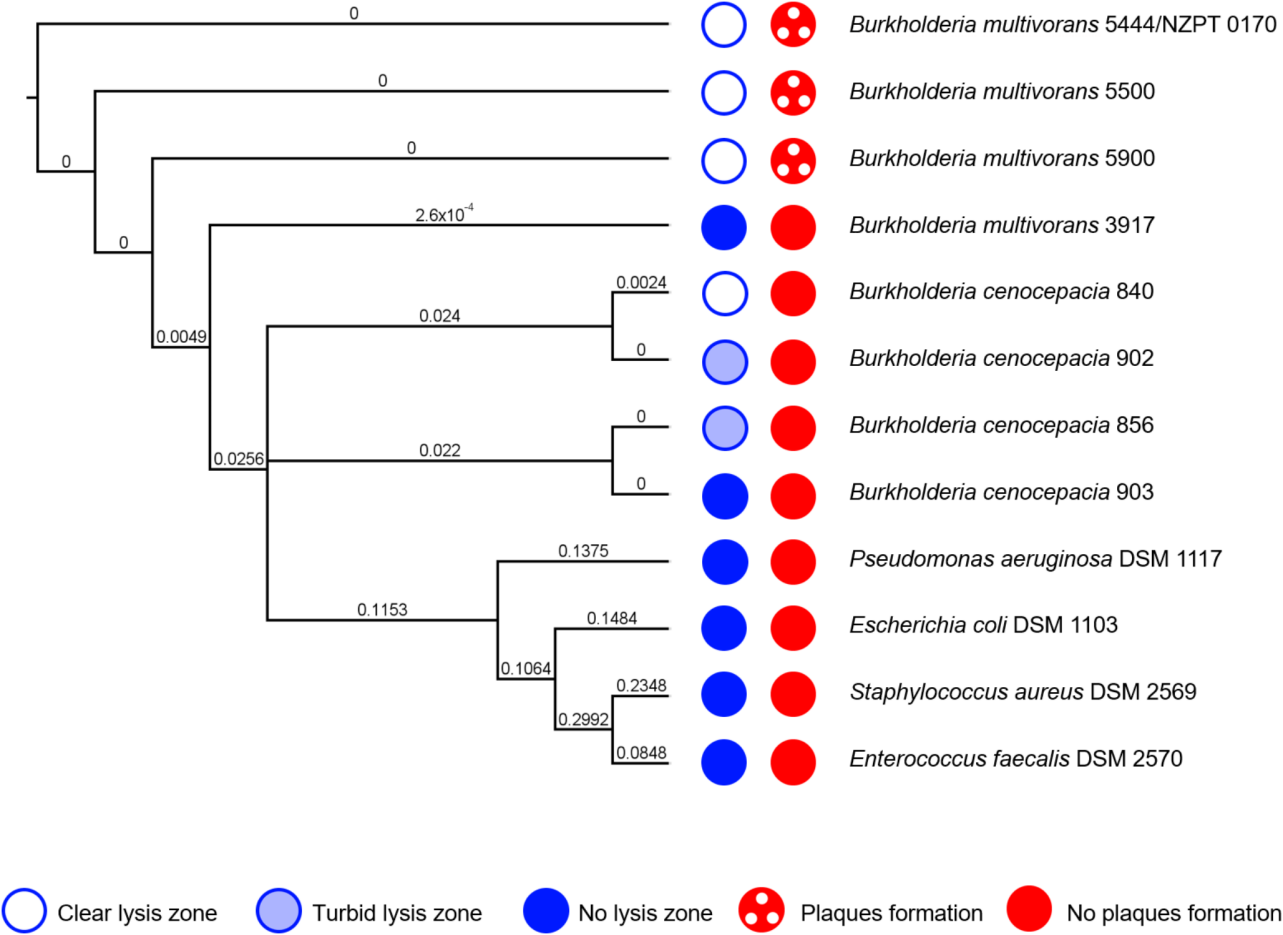
Host range of Bm1, tested using clinical isolates and type strains, whose positions in a phylogenetic tree based on the bacterial *recA* gene are shown. Host range evaluation was performed qualitatively without serial dilution (blue) and quantitatively with serial dilutions (red) of the phage lysate. The phylogenetic tree was generated using proportional transformation, with branch labels representing substitutions per site

Mechanically pre-treated wastewater was used for isolation of a bacteriophage specific for *B. multivorans* strain NZPT 170. Three sequential phage isolation attempts were performed, and a total of 10 wastewater samples were required to obtain a single plaque, which was used to isolate a novel phage, which we named Bm1. In contrast, phages active against bacteria belonging to the order *Enterobacterales* and family *Pseudomonadaceae* predominate in wastewater, and these typically produced dozens of plaques after the first attempt (data not shown). *Burkholderia* phage Bm1 formed clear plaques of 1–2 mm diameter with a tiny halo (Fig. 1). The host range of Bm1 was tested using eight clinical isolates of the *Burkholderia cepacia* complex and four bacterial type strains. In Fig. 2, the results are presented alongside the phylogenetic tree based on the *recA* gene (see also Supplementary Fig. S2). Interestingly, despite its relatively narrow host range, limited to certain *B. multivorans* isolates, phage Bm1 was able to lyse some isolates of *B. cenocepacia* without forming plaques. However, the phylogenetic analysis did not reveal a closer relationship of the isolates from the above-mentioned cystic fibrosis patient to the *B. cenocepacia* isolates than to *B. multivorans* isolate 3917.

### Morphology analysis by TEM

**Fig. 3:**
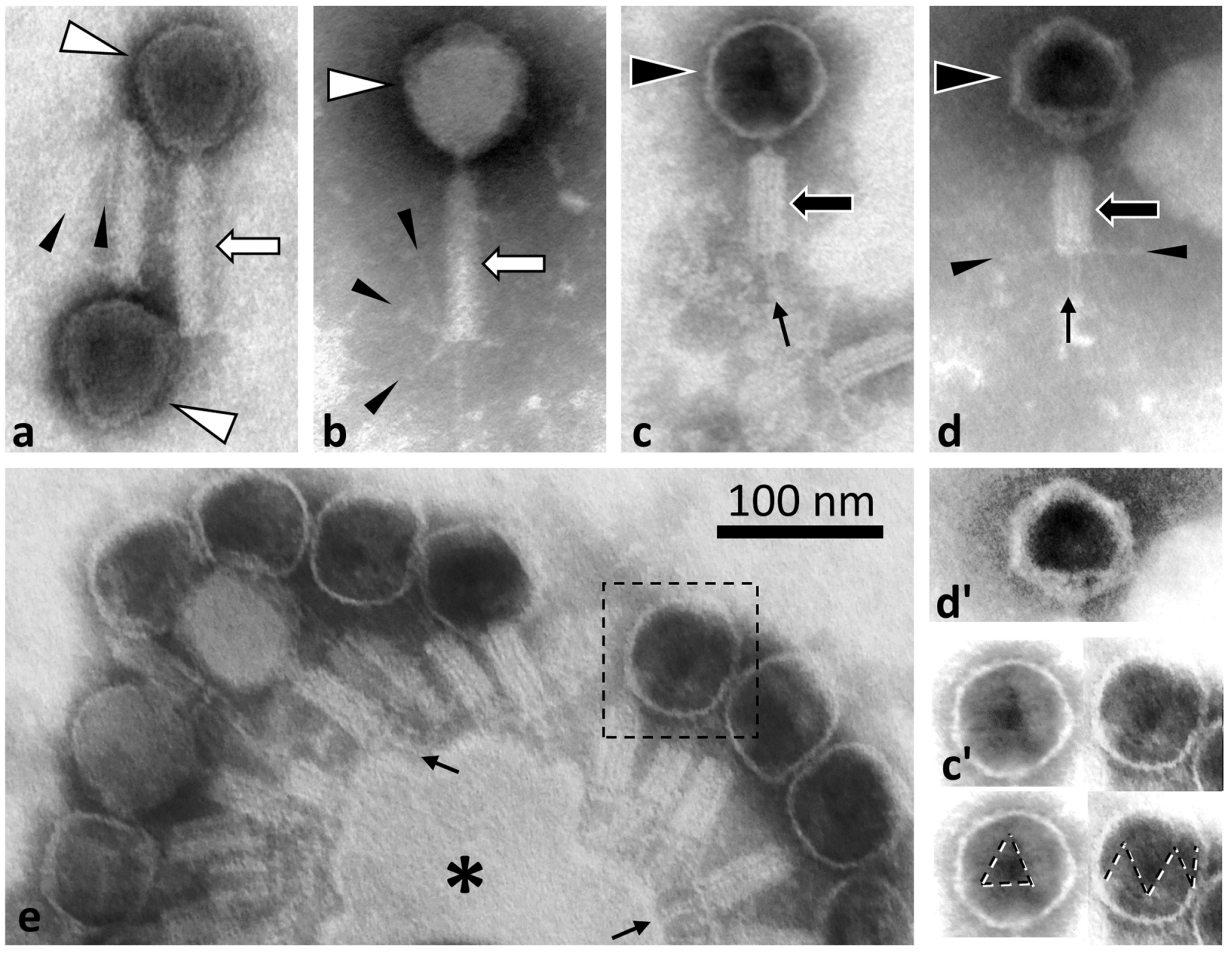
Negative staining transmission electron microscopy of bacteriophages. (**a** and **b**) Phages with an uncontracted sheath. (**c** and **d**) Particles with a contracted sheath and an empty capsid. The capsids in panels **c** and **d** and one in panel e (indicated by a dashed square) are shown with contrast enhancement in panels **c**', **d'**, and **e**'. Also in capsids with a spherical appearance, triangular faces were observed (panels **c**'-**e**'). These are highlighted by dashed lines in duplicate images of panels **c**' and **e**'. In particles with a contracted sheath, the injection tube is visible and is indicated by arrows in panels **c**-**e**. Fibers (small arrowheads) are usually detectable on uncontracted sheaths (**a**, **b**), but sometimes also after contraction (visible in **d**, but not in **c**). Panel **e** shows a group of phages with a contracted sheath and mostly dark capsids, still connected to a cellular remnant (asterisk). The scale bar refers to all images.

Phages were prepared by negative staining and analyzed by transmission electron microscopy (Fig. 3), which revealed a general morphology consisting of a capsid with a tail and fibers. The contractile tail sheath had a length of 100 nm and a width of 18 nm in the uncontracted state and a length of 60 nm and a width of 23 nm in the contracted state (Fig. 3a-d). In particles with a contracted sheath, a tube was visible at the distal end of the sheath (Fig. 3c-e). The cross-diameter of the heads was 72 nm. Triangular faces could be seen in both filled and “ghost” capsids (Fig. 3c' and d'). We also observed cellular remnants in the preparation that were fully covered with phages with contracted sheaths (Fig. 3e)

### One-step growth curve

A one-step growth curve experiment showed that *Burkholderia* phage Bm1 has a 40-min eclipse period, a 60-min latent period, and a burst size of 39 PFU per cell.

**Fig. 4:**
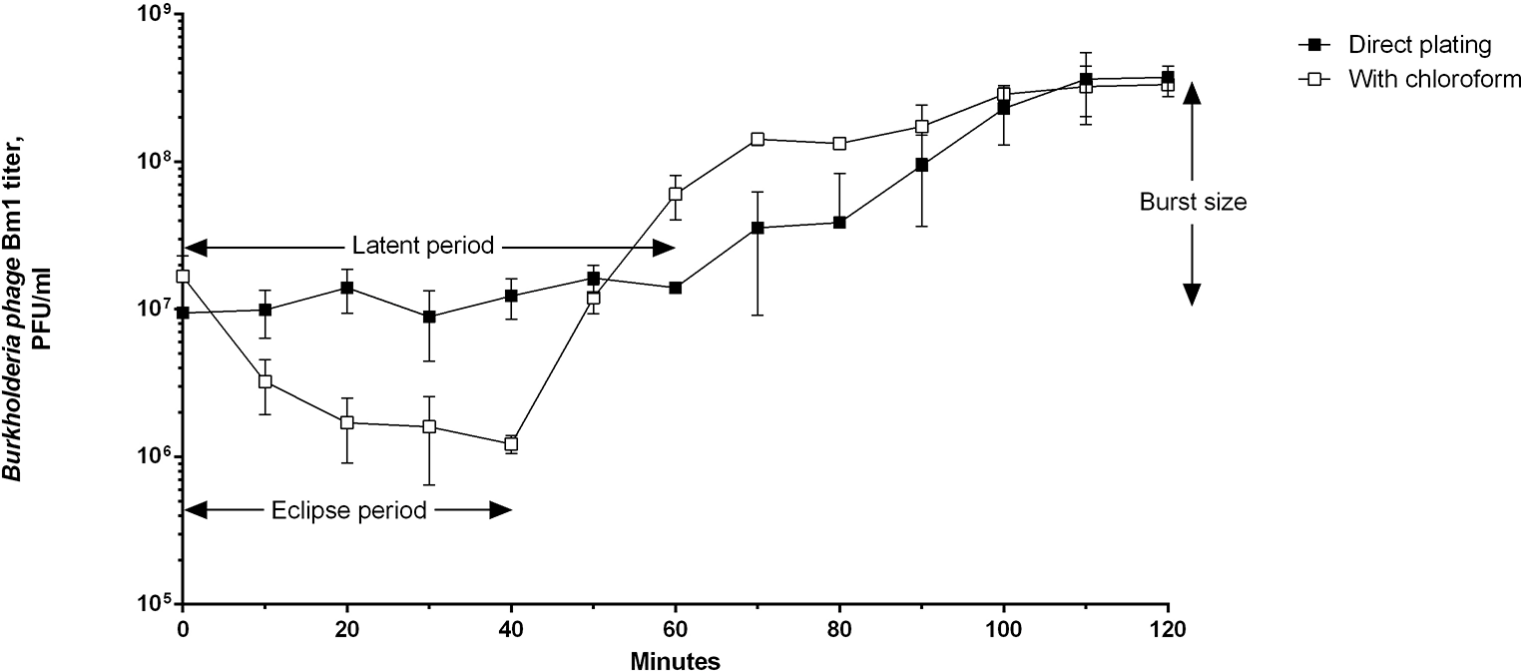
One-step growth curve of *Burkholderia* phage Bm1 using *B. multivorans* 5444 as a host. The direct plating curve represents the number of native infectious centers. The latent period is the period before bacterial cell lysis. The burst size is the ratio of the number of plaques before and after lysis. The "with chloroform" curve represents the total number of bacteriophages including those that were already assembled inside the bacterial cells and released by chloroform treatment. The eclipse period corresponds to the phase in which the bacteriophage exists only in the form of DNA

### Genome sequence and comparative analysis

First, sequencing was performed using the ONT platform, resulting in 110× coverage of the phage genome. No contaminating sequences potentially derived from inducible prophages were detected.

**Fig. 5:**
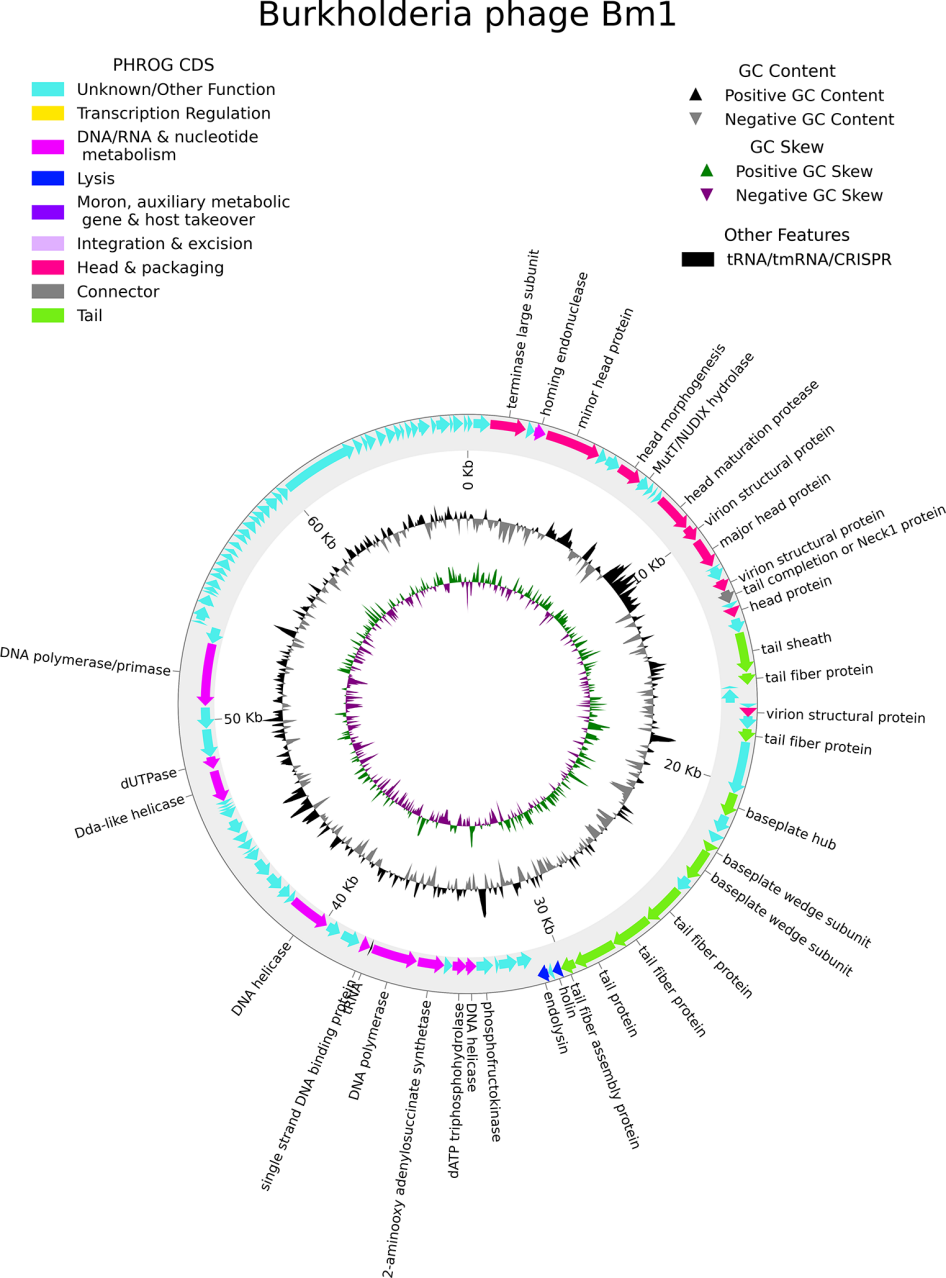
Genomic map of Bm1. The legend shows the symbols and colors for predicted and classified coding sequences, tRNA, GC content, and GC skew

Next, sequencing using an Illumina platform was performed for reconstruction of the complete genome sequence of the phage. The Bm1 genome is a double-stranded DNA of 67,539 bp. The average depth of read coverage was 247×. Manual iterative mapping of the reads to the genome allowed the genomic termini to be resolved, and terminal repeat regions of 359 nucleotides to be identified (Supplementary Figs. S3 and S4). PhageTerm analysis confirmed the presence of redundant genomic termini (left 351 nucleotides) and predicted the phage genome to be circularly permuted with a P1 packaging type (Supplementary Materials). The genome contains 113 genes, 35 of which encode proteins whose function could be predicted based on sequence similarity to known proteins. The remaining 78 gene products were annotated as hypothetical proteins. Of the predicted genes, 79 and 34 were encoded on the forward and reverse DNA strand, respectively. One tRNA gene was predicted on the reverse strand. This tRNA transfers methionine and corresponds to the conventional start codon. The GC content of the phage genome is 56.6%, and its general organization is shown in Fig. 5. Pharokka software integrated tools were used to classify genes and create a genomic map. The genome sequence and its annotation are available in the GenBank database under accession number PP183292.1.

DNA packaging into the bacteriophage head appears to be accomplished with the involvement of a terminase large subunit (CDS_0003). In total, nineteen genes responsible for virion morphogenesis were predicted, included eight head genes, 10 tail genes, and one connective neck protein. A set of 10 genes were predicted to encode two DNA polymerases (CDS_0054 and CDS_0076) and two DNA helicases (CDS_0050 and CDS_0058) involved in DNA replication, recombination, and repair.

Based on its gene content, phage Bm1 appears to use the classical holin (CDS_0043) and endolysin (CDS_0045) pathway for cell lysis. Notably, the endolysin of phage Bm1 has at least 55% amino acid sequence identity to the D-Ala-D-Ala carboxypeptidase family metallohydrolases of a number of members of the genus *Burkholderia*.

Another feature of the bacteriophage Bm1 genome is that it is predicted to encode a MutT protein, or "Nudix" hydrolase (CDS_0010), which provides clearance of potentially harmful endogenous metabolites from the cell and modulates the accumulation of precursors in biochemical pathways [38]. The presence of such an enzyme could potentially influence the rate of accumulation of mutations in the bacteriophage genome. However, the exact function of this gene remains to be elucidated.

**Fig. 6:**
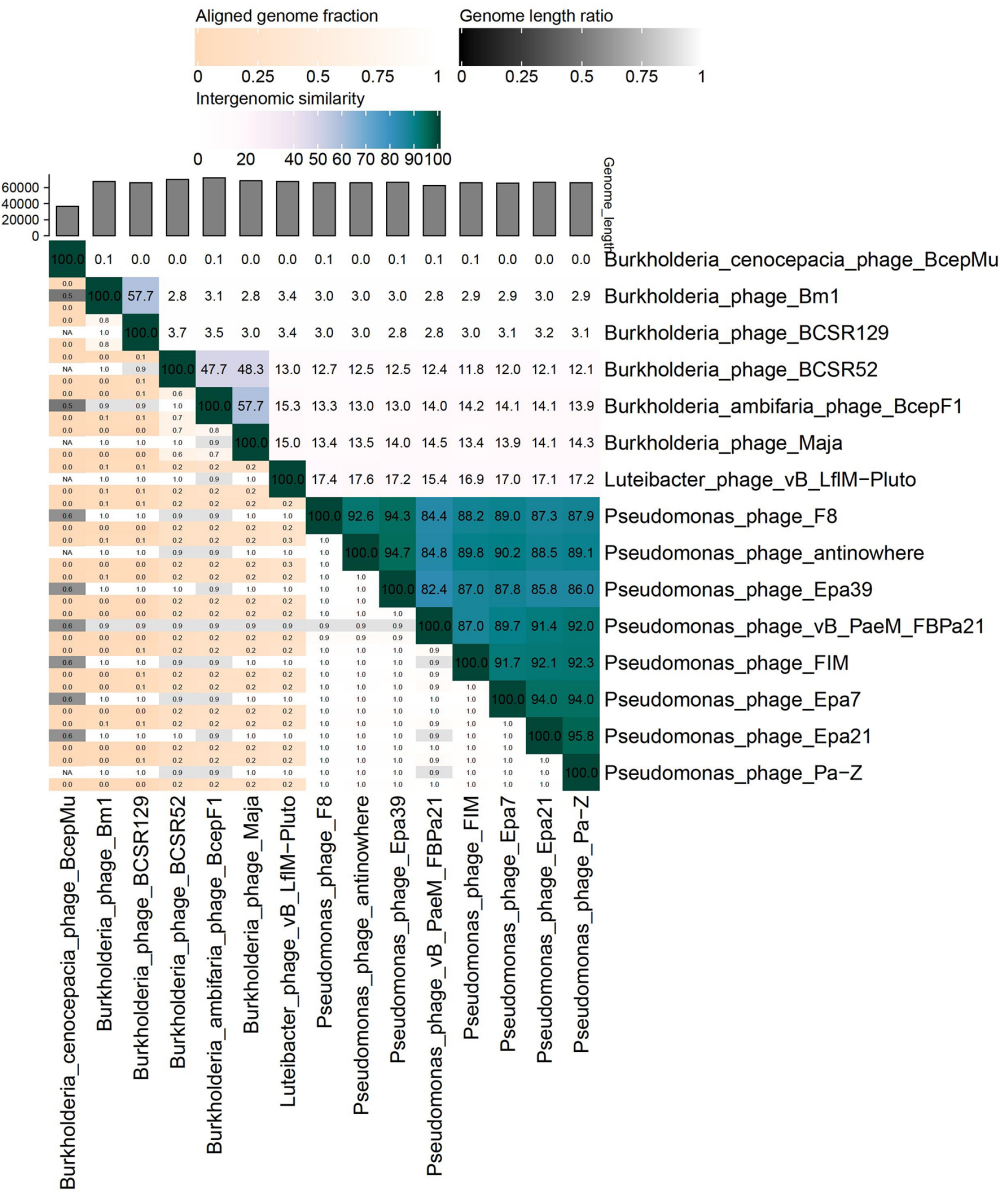
Whole-genome comparison and clustering of phage Bm1 (PP183292.1) with selected bacteriophages. The comparison was performed using VIRIDIC with the default BLASTn parameters. The values represent intergenomic similarity

Comparative analysis of the genome sequence of phage Bm1 using the BLASTn algorithm showed only one bacteriophage to be a close relative, namely the recently described *Burkholderia* phage BCSR129 (GenBank accession no. MW460247.1; query coverage, 48%; identity, 75.58%). Due to the lack of other close relatives, *Burkholderia cenocepacia* phage BcepMu (NC_005882.1), *Burkholderia ambifaria* phage BcepF1 (NC_009015.1), *Burkholderia* phage BCSR52 (MW460246.1), and *Burkholderia* phage Maja (MT708549.1) were selected for comparison using the VIRIDIC calculator. *Luteibacter* phage vB_LflM-Pluto (ON529861.1) was also included due to the relatively high degree of amino acid sequence similarity of its proteins to the major head protein (CDS_0016) and tail sheath protein (CDS_0024) of phage Bm1 (63% and 50% identity, respectively). *Pseudomonas* phages were also included as examples of virulent phages with similar virion morphology (Fig. 6).

It was confirmed that phage Bm1 has the highest intergenomic similarity to phage BCSR129 (57.7%). This is below the currently accepted threshold of 70% for assigning bacteriophages to the same genus [39].

We did not detect any genes in the phage Bm1 genome that were similar to known integrases or any other genes associated with a temperate life cycle. An analysis using the PhageAI platform predicted a virulent type with 98.06% probability.

## Conclusions

Strictly lytic (virulent) bacteriophages are an important component of the fight against antibiotic resistance, as they are the best suited for phage therapy. The novel, presumably virulent, *Burkholderia* phage Bm1 presented here is lytically active against a pan-drug-resistant *Burkholderia multivorans* isolate from a cystic fibrosis patient. The characteristics of this phage make it a promising candidate for phage therapy applications. In addition, its genetic divergence from other known phages suggests that it should be classified as a member of a new genus.

## Electronic Supplementary Material

Below is the link to the electronic supplementary material

Supplementary Material 1
