# Supplementary material for: Characterization and genome analysis of the novel virulent Burkholderia phage Bm1, which is active against pan-drug-resistant Burkholderia multivorans
Source: Arch Virol. 2025 Apr 16;170(5):106. doi: 10.1007/s00705-025-06282-w (PMC12003559; doi:10.1007/s00705-025-06282-w)
Supplement: Supplementary file 1 — Supplementary Material 1 [file 705_2025_6282_MOESM1_ESM.docx]

Supplementary materials

Characterization and genome analysis of novel virulent Burkholderia phage Bm1 active against pan-drug resistant Burkholderia multivorans

**Evgenii Rubalskii^1,2,^*,** **Ludwig Sedlacek^3^, Jan Hegermann^4^, Leonard Knegendorf^3^, Christina Salmoukas^1,2^, Carsten Mueller^5^, Nicolaus Schwerk^5^, Dirk Schlüter^3^, Arjang Ruhparwar ^1,2^, Christian Kuehn ^1,2^, Stefan Ruemke ^1,2^**


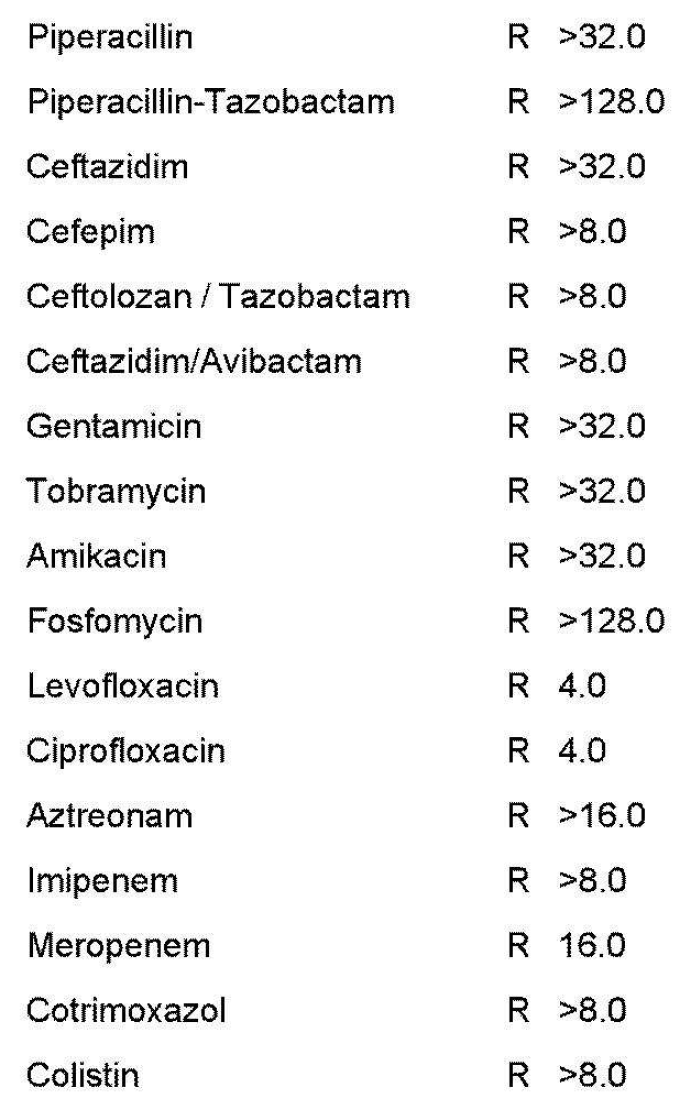


Figure S1. **Antibiotic susceptibility of *B. multivorans* 5444 (NZPT 0170) determined by MIC method.**


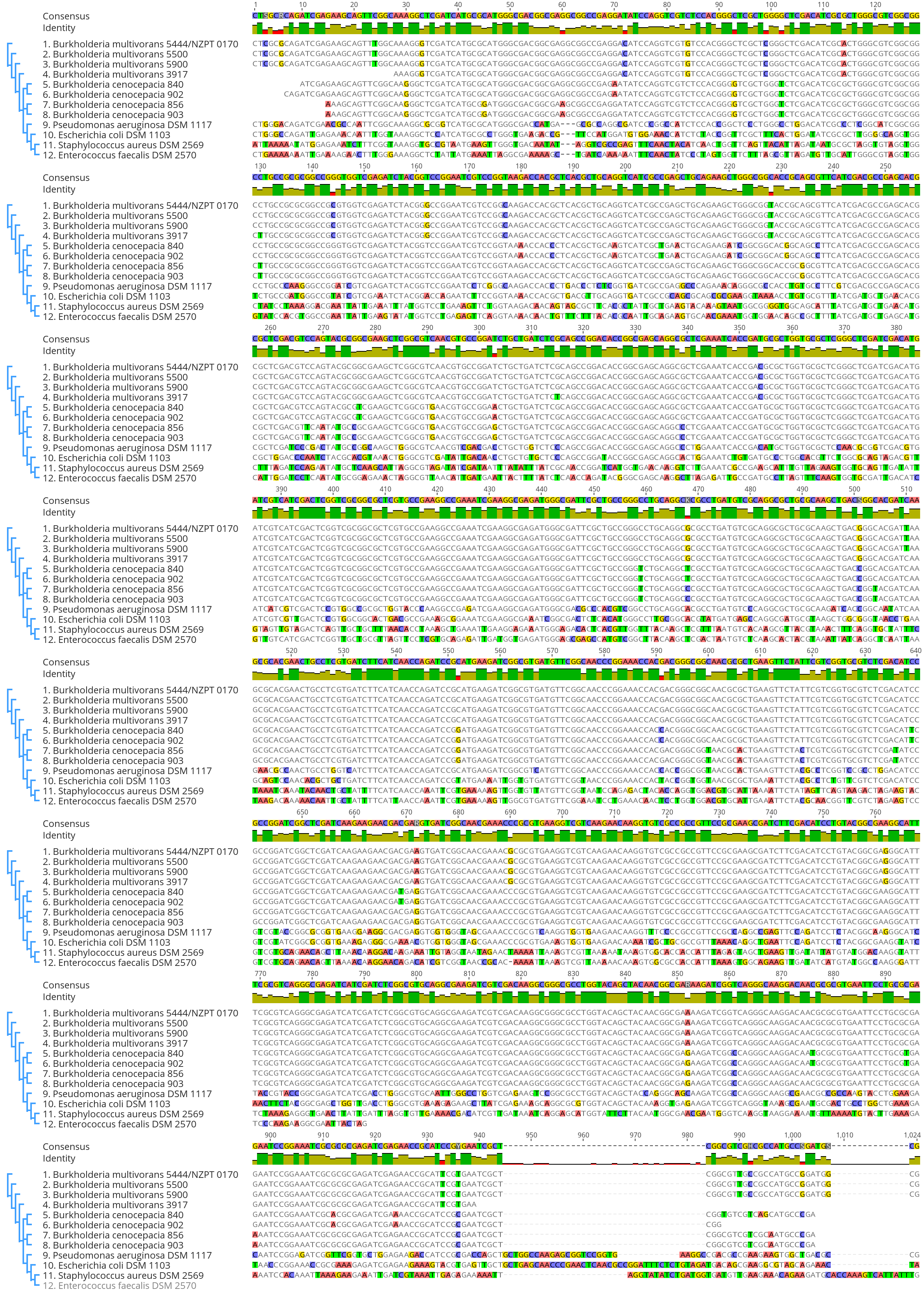


Figure S2. **Multiple nucleotide alignment the *recA* gene.**


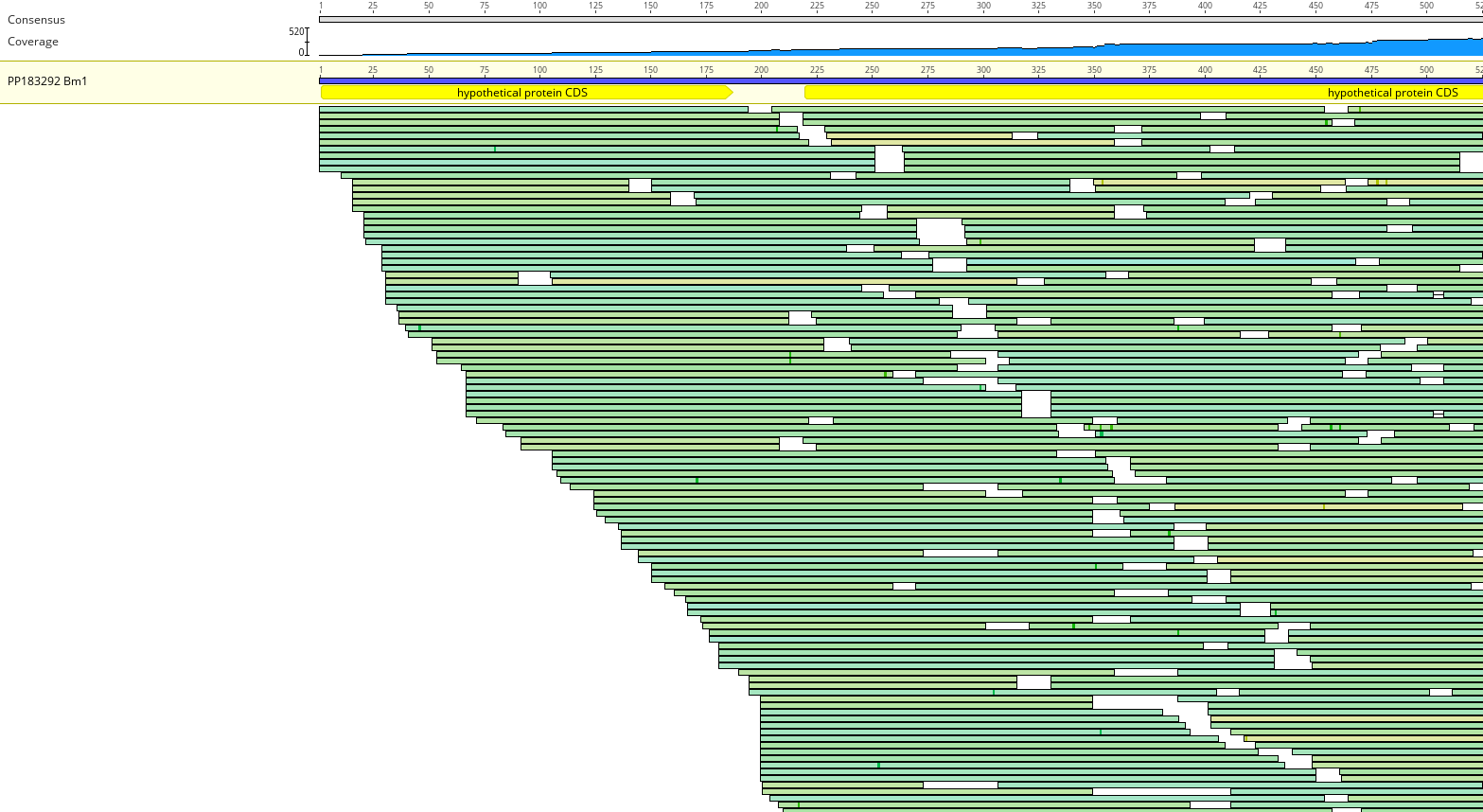
Figure S3. **Mapping of Illumina paired reads to left terminal genomic region of the *Burkholderia* phage Bm1.**


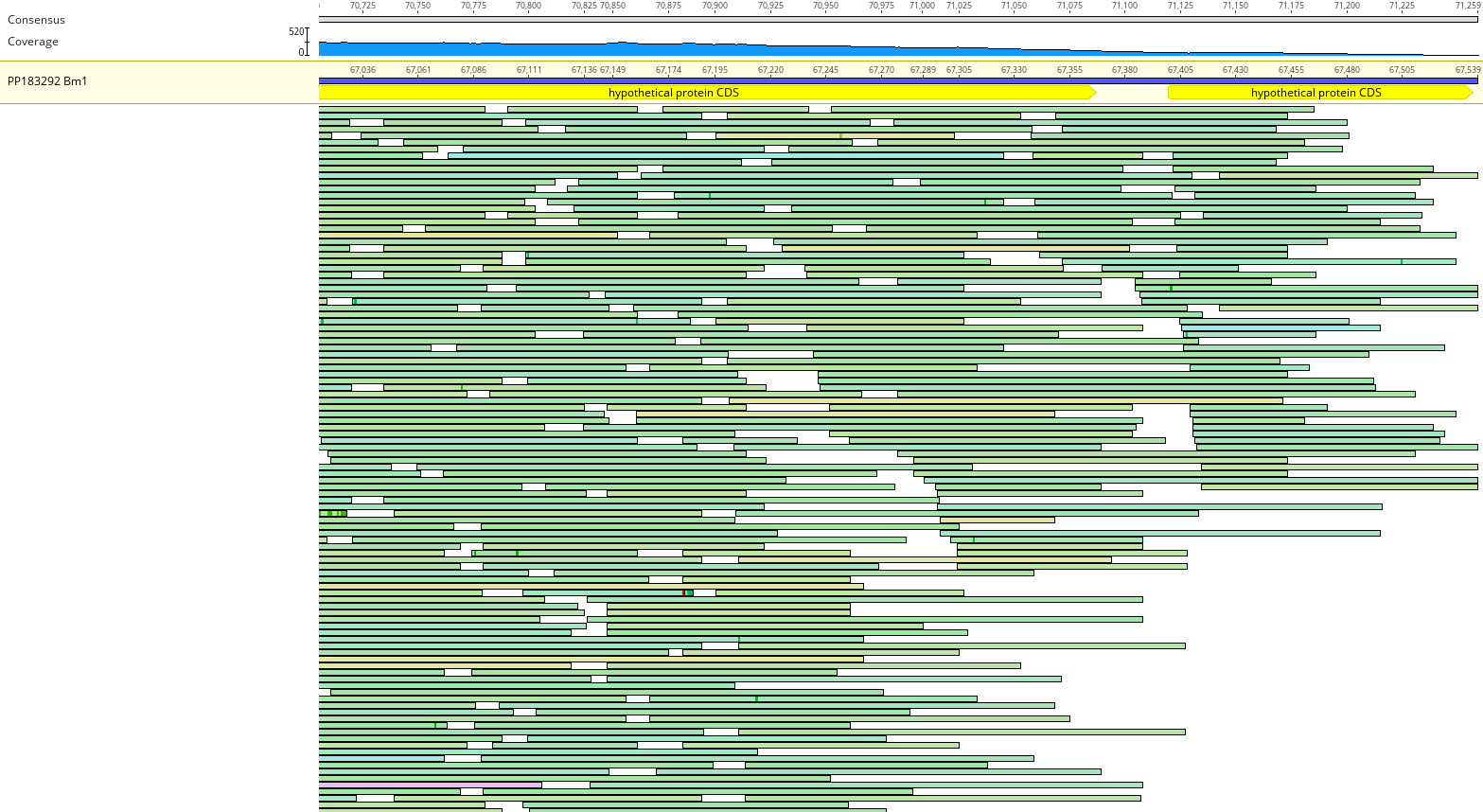


Figure S4. **Mapping of Illumina paired reads to right terminal genomic region of the *Burkholderia* phage Bm1.**


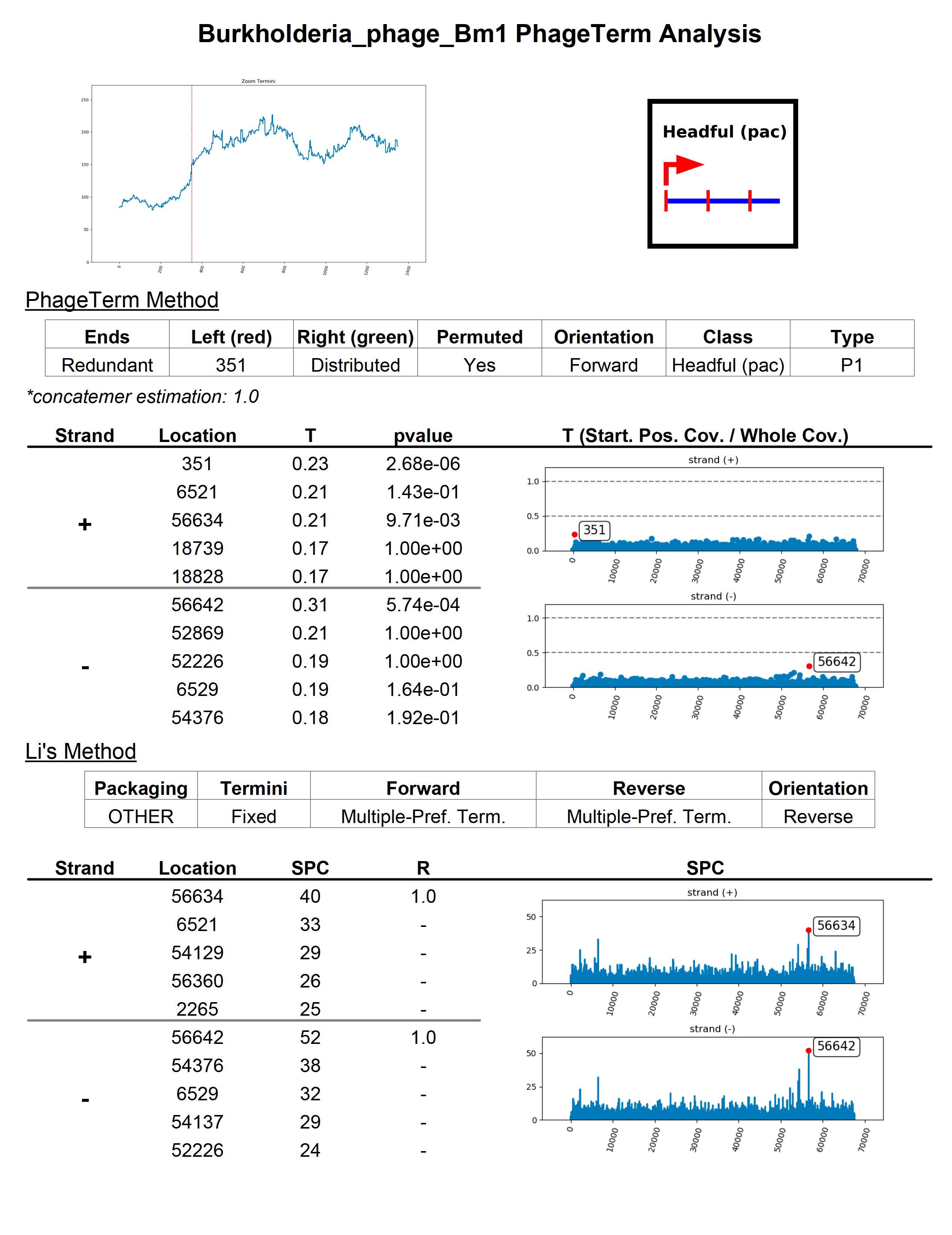


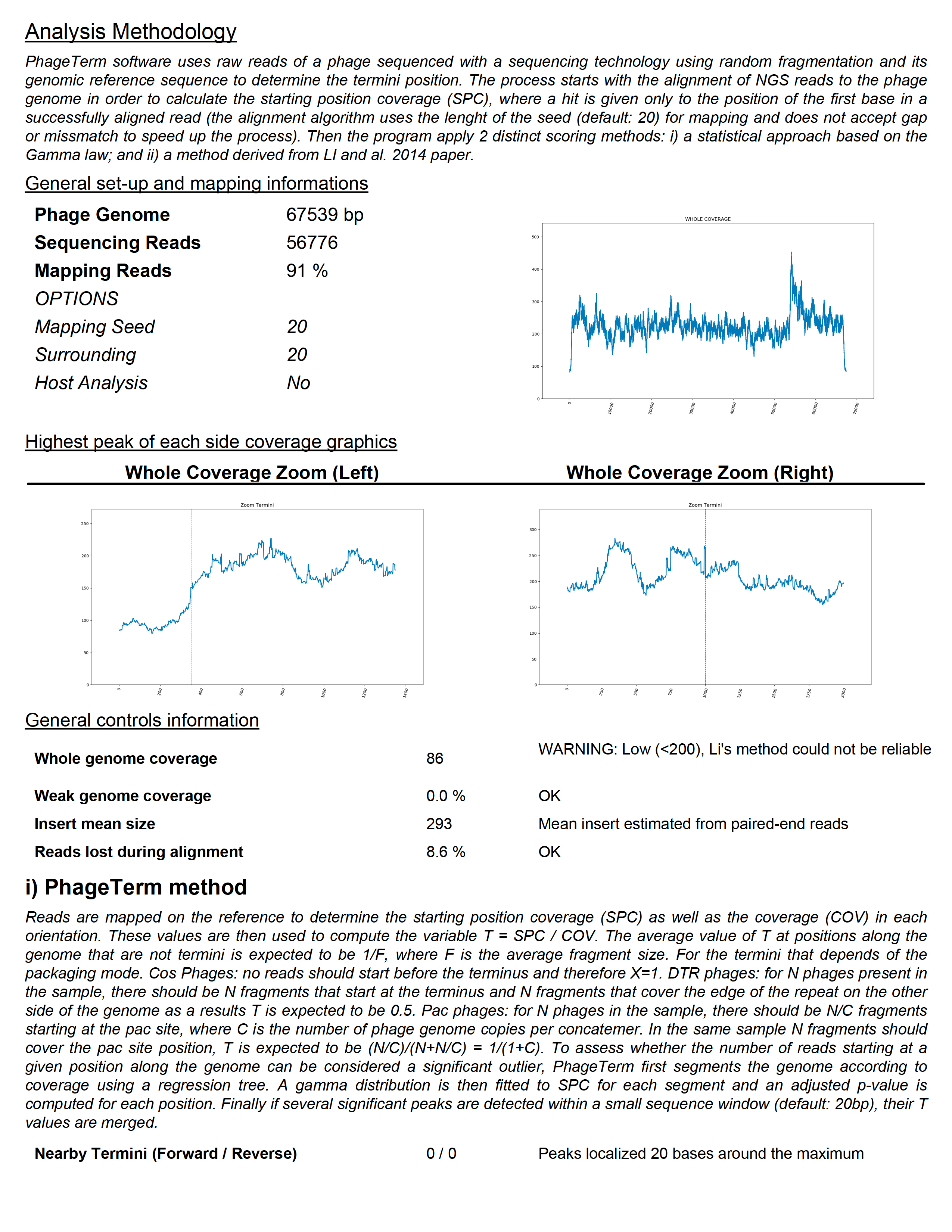


Table S1. **Plaque counts and average parameters of one-step growth curve of *Burkholderia* phage Bm1 on host *B. multivorans* 5444**

|  |  | **Concentration of Burkholderia phage Bm1, PFU/mL** | | | | | | |
| --- | --- | --- | --- | --- | --- | --- | --- | --- |
|  |  | **Minutes** | **Direct plating** | | | **With chloroform** | | |
|  |  |  | **n1** | **n2** | **n3** | **n1** | **n2** | **n3** |
| Eclipse period | Latent period | 0 | 9,00E+06 | 1,03E+07 | 9,00E+06 | 2,40E+07 | 1,30E+07 | 1,30E+07 |
|  |  | 10 | 8,00E+06 | 7,70E+06 | 1,40E+07 | 3,10E+06 | 4,60E+06 | 2,00E+06 |
|  |  | 20 | 9,00E+06 | 1,80E+07 | 1,50E+07 | 1,40E+06 | 2,60E+06 | 1,10E+06 |
|  |  | 30 | 1,40E+07 | 7,00E+06 | 5,70E+06 | 7,00E+05 | 2,60E+06 | 1,50E+06 |
|  |  | 40 | 8,00E+06 | 1,40E+07 | 1,50E+07 | 1,29E+06 | 1,03E+06 | 1,35E+06 |
|  |  | 50 | 1,30E+07 | 1,60E+07 | 2,00E+07 | 9,00E+06 | 1,30E+07 | 1,40E+07 |
|  |  | 60 | 1,30E+07 | 1,50E+07 | 1,40E+07 | 6,30E+07 | 3,90E+07 | 7,90E+07 |
|  |  | 70 | 3,40E+07 | 1,00E+07 | 6,30E+07 | 1,27E+08 | 1,46E+08 | 1,53E+08 |
|  |  | 80 | 9,00E+07 | 1,55E+07 | 1,05E+07 | 1,20E+08 | 1,40E+08 | 1,40E+08 |
|  |  | 90 | 1,42E+08 | 3,00E+07 | 1,11E+08 | 2,50E+08 | 1,20E+08 | 1,50E+08 |
|  |  | 100 | 1,32E+08 | 2,25E+08 | 3,31E+08 | 2,80E+08 | 3,20E+08 | 2,60E+08 |
|  |  | 110 | 1,80E+08 | 3,60E+08 | 5,50E+08 | 4,40E+08 | 2,00E+08 | 3,30E+08 |
|  |  | 120 | 3,60E+08 | 3,10E+08 | 4,50E+08 | 3,50E+08 | 3,80E+08 | 2,70E+08 |
|  |  |  |  |  |  |  |  |  |
|  |  | **Average parameters** | | | | | |  |
|  |  | Adsorption control (AC) | | | | 3,23E+06 | PFU/mL |  |
|  |  | Latent line (LL) | | | | 1,26E+07 | PFU/mL |  |
|  |  | Number of infected cells (IC) = LL - AC | | | | 9,34E+06 | cells |  |
|  |  | Lysis plateau (LP) | | | | 3,68E+08 | PFU/mL |  |
|  |  | **Average burst size (ABZ) = LP / IC** | | | | **39** | **PFU/cell** |  |
